# Does sleep matter for recovery after stroke? Functional severity shapes the association between sleep and functional outcome during inpatient stroke rehabilitation

**DOI:** 10.1007/s10072-026-09374-w

**Published:** 2026-10-01

**Authors:** Serena Scarpelli, Valentina Alfonsi, Maurizio Gorgoni, Erika Di Matteo, Natasha Manili, Ludovica Annarumma, Elisa Pellegrini, Camilla Mattei, Giovanni Morone, Mariella Pazzaglia, Luigi De Gennaro, Marco Iosa

**Affiliations:** 1 Department of Psychology, Sapienza University of Rome, Via dei Marsi 78, 00185 Rome, Italy; 2 IRCCS Fondazione Santa Lucia, Rome, Italy; 3 Departmental Faculty of Medicine and Surgery, UniCamillus-Saint Camillus International University of Health Sciences, Rome, Italy; 4 Department of Life, Health and Environmental Sciences, University of L’Aquila, L’Aquila, Italy

**Keywords:** Actigraphy, Sstroke, Rehabilitation, Barthel index, Sleep duration

## Abstract

**Background:**

Sleep disturbances are common after stroke and have been associated with rehabilitation outcomes. The clinical meaning of post-stroke sleep duration remains unclear, as longer sleep may be associated with different clinical and functional states, including greater disability. This exploratory study investigated the relationship between sleep and functional outcome during stroke rehabilitation, and the moderating role of baseline functional status.

**Methods:**

Twenty-nine patients with stroke underwent a 10-day longitudinal monitoring protocol during rehabilitation. Functional independence and motor performance were assessed at the beginning (T0) and the end (T1) of the observation period using the Barthel Index, 10-Meter Walk Test, and 9-Hole Peg Test. Sleep was assessed throughout the monitoring period using sleep diaries, and actigraphy .

**Results:**

Functional independence improved from T0 to T1, whereas motor performance did not significantly change. In unadjusted analyses, longer actigraphy-derived total sleep time and longer self-reported total time in bed were correlated with lower Barthel Index scores at T1. Moderation analyses adjusted for prior rehabilitation days showed that longer total sleep time was positively associated with Barthel Index at T1 only in patients with higher baseline independence, while longer time in bed was positively associated at average and higher baseline levels.

**Conclusions:**

The associations between sleep duration or time in bed and later post-rehabilitation functional independence vary according to baseline functional status. Given the small sample size and observational design, these findings should be interpreted cautiously and considered preliminary and hypothesis-generating.

**Supplementary Information:**

The online version contains supplementary material available at https://doi.org/10.1007/s10072-026-09374-w.

## Introduction

Sleep is a fundamental biological process for neurological health, with a key role in cognition, learning, and experience-dependent plasticity [1, 2]. After brain injury, sleep is increasingly considered an active condition supporting memory consolidation and neural circuit reorganization, processes that are highly relevant to post-stroke recovery [1, 2]. Accordingly, sleep has been investigated both as a factor associated with cerebrovascular risk and in relation to rehabilitation outcomes after stroke [3].

A growing body of literature supports a bidirectional relationship between sleep and stroke. Sleep disturbances and sleep disorders may increase stroke risk, while stroke itself can disrupt sleep duration, continuity, architecture, and circadian regulation [3–6]. Beyond sleep-disordered breathing [3], stroke survivors often report insomnia symptoms, fragmented sleep, altered sleep duration, reduced sleep efficiency, and poorer subjective sleep quality, all of which have been associated with functional status and rehabilitation engagement [7–10]. Importantly, sleep is a multidimensional construct, and different objective and subjective measures may reflect distinct aspects of post-stroke condition. Sleep efficiency reflects the continuity and consolidation of sleep. A systematic review and meta-analysis of polysomnographic studies showed that stroke survivors commonly exhibit reduced sleep efficiency, together with increased wakefulness after sleep onset and greater sleep fragmentation, compared with healthy controls [11]. These alterations indicate impaired sleep continuity, which may compromise the restorative functions of sleep. Subjective sleep quality, in contrast, reflects the patient’s overall perception of how satisfactory and restorative sleep has been and may not necessarily correspond to objective sleep measures. Nevertheless, poorer subjective sleep quality has been associated with worse functional status and motor performance in patients undergoing stroke rehabilitation [12], suggesting that patients’ perception of sleep may provide clinically relevant information beyond objective sleep parameters. Finally, time in bed reflects the opportunity for sleep rather than the amount of sleep itself, as it includes both sleeping and waking periods. In patients undergoing stroke rehabilitation, prolonged time in bed may reflect functional dependency, reduced mobility, lower daytime activity, and environmental constraints associated with inpatient care, in addition to sleep behavior [13, 14]. Consequently, time in bed should not be interpreted interchangeably with sleep duration, as these measures may capture different aspects of the patient’s clinical condition.

Sleep duration has received particular attention, as both short and long sleep have been associated with stroke incidence and mortality, with stronger associations often reported for long sleep [15, 16]. However, the interpretation of sleep duration after stroke remains complex. Short sleep may be associated with less favorable restorative and plasticity-related conditions, whereas long sleep may reflect either adaptive neural processes or greater disability, reduced daytime activation, and broader clinical burden [6, 8, 10, 11].

This complexity is especially relevant in inpatient rehabilitation, where sleep has been studied in relation to learning and plasticity, but may also be influenced by inactivity, medical burden, neurological severity, and environmental factors such as shared rooms, clinical care routines, light exposure, and noise [1, 8, 17, 18]. Baseline functional severity may therefore be a crucial but often overlooked factor, as more impaired patients may sleep longer, spend more time in bed, or report poorer sleep because of greater disability at admission.

Against this background, we conducted an exploratory study longitudinally investigating sleep during inpatient stroke rehabilitation using both objective actigraphy and subjective sleep measures. Specifically, the study aimed to: (a) evaluate functional progress during rehabilitation by assessing changes in functional independence and motor performance alongside sleep monitoring; (b) explore associations between sleep parameters and rehabilitation-related variables that significantly improved over time; and (c) test whether functional severity at the start of the observation period moderated the association between sleep parameters and functional status at T1, helping to clarify whether these associations varied according to initial functional status [6, 9].

## Method

### Participants

The present study was conducted on a total of 29 patients diagnosed with stroke (hemorrhagic = 7; ischemic = 22) admitted as inpatients at the IRCCS Santa Lucia Foundation in Rome. The characteristics of the sample are detailed in Table 1.

**Table 1:** Characteristics of the sample

	M (SD) *N* = 29
Sex (Males/Females)	21/8
Age (years)	65.6 (10.5)
Stroke side (Right/Left/Bilateral):	12/16/1
Stroke type (Hemorrhagic/Ischemic)	7/22
Time since stroke (days)	68.6 (50.8)
Days of rehabilitation completed	45.8 (39.7)

Of the 29 patients who participated in the experimental protocol, 21 were male and 8 were female, with mean age 65.6 ± 10.5 years. Regarding the side of the lesion, 12 patients presented right hemisphere involvement, 16 left hemisphere involvement, and 1 bilateral involvement. The time interval since the vascular event was on average 68.6 days, while the rehabilitation period averaged 45.8 days.

Patients involved in the study had to meet the following inclusion criteria: (1) a diagnosis of stroke confirmed by neuroimaging; (2) admission in the subacute phase at the IRCCS Santa Lucia Foundation. Patients were excluded from the study if they presented: (1) a score lower than 24 on the Mini-Mental State Examination (MMSE; [19]), a screening battery used to examine global cognitive functioning; (2) lack of adequate linguistic skills to understand the questions; (3) a confirmed previous sleep disorders due to OSAS (Obstructive Sleep Apnea Syndrome); (4) severe comorbidities related to other neurological pathologies.

### Study design

The study involved the longitudinal collection of experimental data over a 10-day period (two weeks excluding weekends) while the subacute stroke patients were undergoing rehabilitation. For each participant, demographic and clinical information was collected at T0 (age, stroke type, lesion hemisphere, time since stroke). Furthermore, the Pittsburgh Sleep Quality Index (PSQI; [20]) was utilized to retrospectively assess sleep quality for each participant at the start of data collection (T0).

At the beginning (T0) and end (T1) of the 10-day experimental procedure, measurements were taken for functional independence (Barthel Index [21]), and motor performance (9-Hole Peg Test – 9HPT; [22], and 10-Meter Walk Test – 10MWT; [23]).

Throughout the 10-day monitoring period, subjects wore a wrist-worn actigraph on the non-paretic arm to obtain objective sleep indices and completed a sleep diary within 30 min of awakening. The latter measure was collected every morning by the experimenter to assist the patient. Furthermore, at the start of each initial rehabilitation session, the patient’s level of participation was recorded by the therapist using the Pittsburgh Rehabilitation Participation Scale (PRPS; [24]. Longitudinal measures were collected for a total of 10 consecutive days, accounting for the suspension of rehabilitation activities during weekends. Also, participants underwent overnight screening with pulse oximetry (at least one night) according to routine clinical practice before study inclusion. No cases of clinically overt obstructive sleep apnea were identified.

A detailed description of each instrument used is provided in the Supplementary Material.

Participants could only take part in the protocol after reading and signing the informed consent. The data used were anonymized with an alphanumeric code. Participation was voluntary, with the right to freely withdraw from the study without data retention and was not associated with any financial compensation. The study was conducted with the approval of the Ethics Committee of Santa Lucia Foundation (Prot. CE/2022_001) and in accordance with the Declaration of Helsinki.

### Statistical analyses

All statistical analyses were performed using SPSS 28.0, Jamovi 2.3.28 and MATLAB 2020. P-values were set < 0.05.

### Preliminary and control analyses

Two control analyses were conducted and are provided in the Supplementary Materials. Specifically, a first control analysis was performed to verify that “good” and “poor” sleepers at T0, as assessed through the PSQI, did not differ in terms of functional improvement, motor performance, or objective and subjective sleep measures collected during the 10-day target period. Second, an ANOVA for repeated measures was conducted on the PRPS scores across the 10-day rehabilitation period. This analysis was intended to ensure that participation remained stable throughout the target period, suggesting that functional gains were not biased by fluctuations in patient engagement.

### Changes in functional independence and motor performance

To evaluate changes in functional independence (Barthel Index) and motor performance (9HPT, 10MWT) from T0 to T1, a series of repeated measures ANCOVAs were performed. This approach allowed the assessment of clinical improvement while explicitly controlling for baseline differences in rehabilitation exposure by including, as a covariate, the total number of rehabilitation days already completed at the beginning of data collection.

Differences in sample size across analyses reflect task-specific feasibility, as a subset of patients was unable to complete certain motor assessments at baseline (e.g., walking or manual dexterity tasks). To ensure within-subject comparability, analyses were restricted to participants with complete data at both time points (T0 and T1) for each outcome.

### Correlation analyses

Exploratory correlations (Pearson’s r or Spearman’s rho, according to the normality or not of data distributions) were conducted to examine the associations between both objective (actigraphy) and subjective (diary) sleep parameters and functional outcomes at T1. Only outcomes resulting significantly different in the previous statistical comparisons were included in the correlation analyses in the main manuscript; correlations with other parameters were reported in Supplementary Materials.

Subjective and actigraphy-derived total bed time (TBT) represent the same time interval, because the actigraphy scoring window was defined on the basis of the self-reported bedtimes and get-up times recorded in the sleep diary.

Given the exploratory nature of these analyses and the limited sample size, no formal correction for multiple comparisons was applied. The resulting associations were therefore considered preliminary and were used to identify candidate sleep variables for subsequent exploratory moderation analyses.

### Moderation analyses

Moderation analyses were performed to further investigate the relationship between sleep and final outcomes based on significant correlations we found in previous analyses. Specifically, based on the hypothesis that the initial functional impairment of patients may influence the relationship between sleep and outcome, in each model, Barthel Index at T1 was entered as the dependent variable, the sleep parameter was entered as the predictor, and Barthel Index at T0 was entered as a continuous moderator. Thus, Barthel Index at T0 was included both as a main effect and in the interaction term with the sleep variable. This approach tested whether the association between sleep and Barthel Index at T1 varied across the continuous range of baseline functional independence, while accounting for baseline Barthel Index, rather than comparing predefined severity groups or directly modelling change scores. Separate models were estimated for actigraphy-derived total sleep time (TST) and TBT. The number of rehabilitation days completed before the beginning of the monitoring period was additionally included as a covariate to account for differences in prior rehabilitation exposure. Significant sleep × Barthel Index at T0 interactions were further examined using simple slope analyses at low (− 1 SD), mean, and high (+ 1 SD) levels of baseline functional independence.

## Results

### Preliminary and control analyses

Results of preliminary control analyses were reported in Supplementary Materials, revealing no differences between poor and good sleepers, as well as no significant changes in participation across the 10days of data collection.

### Functional and motor improvement

Regarding measures of functional independence (*N* = 29), the ANCOVA revealed a statistically significant main effect of TIME, even controlling for the number of rehabilitation days (F_(1, 27)_ = 18.802, *p*<.001, ηp²=0.411). Indeed, mean Barthel Index scores increased from T0 (M = 51.72 ± 23.04) to T1 (M = 60.00 ± 24.93). The interaction between TIME and the number of rehabilitation days was not significant (F_(1, 27)_ = 2.299, *p* = .141, ηp² =0.078), and the covariate itself was not a significant predictor of Barthel scores (F_(1, 27)_ = 1.407, *p*=.246, ηp²=0.050).

Concerning motor performance assessed with the 10MWT, twelve patients were included in the analysis (*N* = 12). A descriptive reduction in walking time was observed from T0 (M = 27.79 ± 23.58 s) to T1 (M = 21.87 ± 20.14 s). However, after including the number of rehabilitation days as a covariate, the main effect of TIME was not statistically significant (F_(1, 10)_ = 1.777, *p* = .212, ηp² =0.151). The interaction between TIME and rehabilitation days was not significant (F_(1, 10)_ = 0.135, *p*=.721, ηp²=0.013). In this model, the number of rehabilitation days was a significant predictor of walking performance (F_(1, 10)_ = 5.865, *p*=.036, ηp²=0.370).

Concerning statistical comparisons for 9HPT paretic arm, fourteen patients were included in the analysis (*N* = 14). The main effect of TIME was not statistically significant (M = 33.99 ± 19.69 s at T0; M = 31.56 ± 12.47 s at T1; F_(1, 12)_ = 0.698, *p*=.420, ηp²=0.055). The TIME × rehabilitation days interaction was not significant (F_(1, 12)_ = 0.216,*p*=.650, ηp²=0.018), and the covariate was not significant (F_(1, 12)_ = 0.006, *p*=.938, ηp²=0.001).

Regarding the non-paretic arm 9HPT, twenty-seven patients were included in the analysis (*N* = 27). The main effect of TIME did not reach statistical significance (M = 23.97 ± 9.03 s at T0; M = 22.41 ± 7.25 s at T1; F_(1, 25)_ = 3.717, *p*=.065, ηp²=0.129). The TIME × rehabilitation days interaction was not significant (F_(1, 25)_ = 2.102, *p*=.159, ηp²=0.078), and the covariate was not significant (F_(1, 25)_ = 0.089, *p*=.768, ηp²=0.004).

**Fig. 1:**
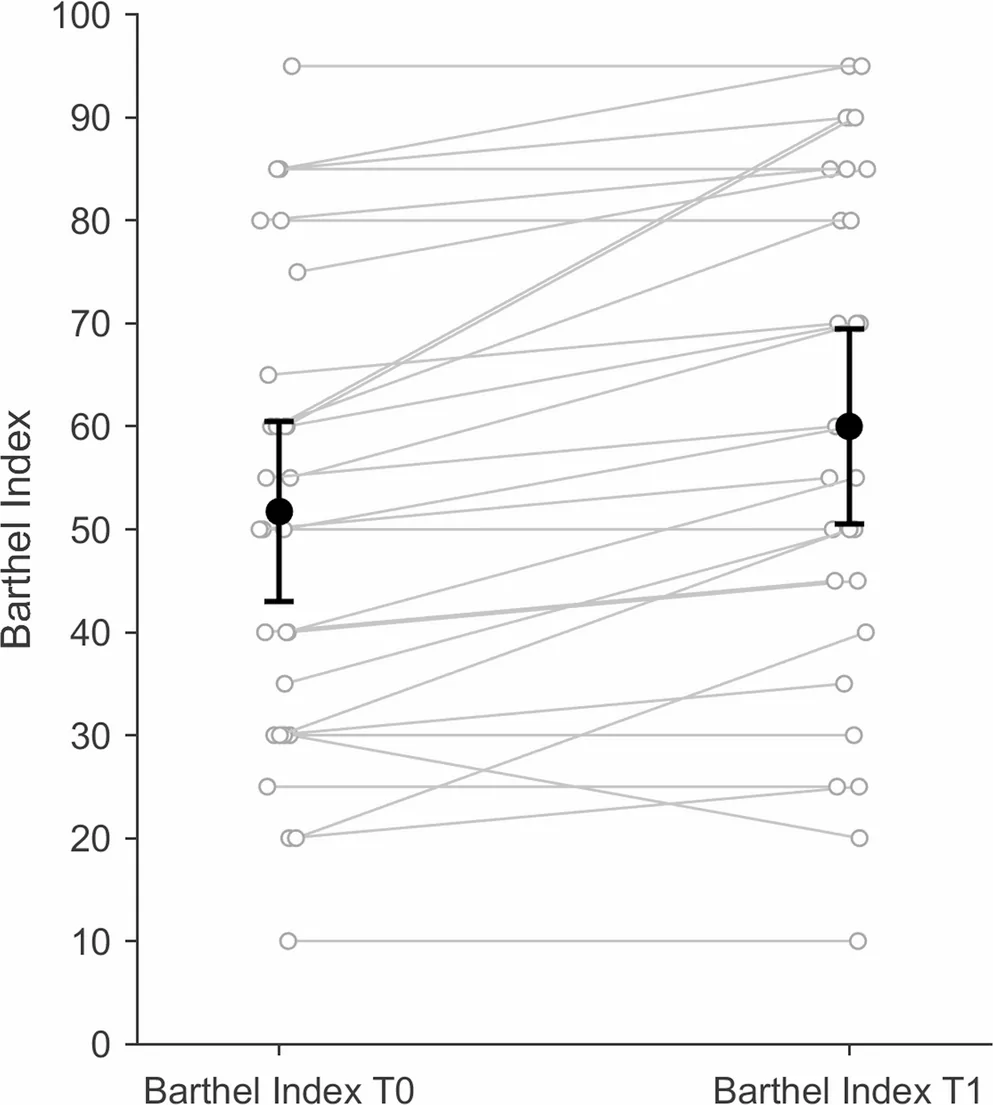
Individual changes in Barthel Index between T0 and T1. Thin grey lines connect repeated measurements from the same participant. Black circles indicate group means and error bars represent 95% confidence intervals

ANCOVA results are reported in Table 2, while the individual trajectories underlying the significant pre–post improvement in the Barthel Index are illustrated in Fig. 1.

**Table 2:** Effects of time (T0 vs T1) on functional and motor outcomes controlling the number of rehabilitation days

Outcome	N	T0Mean± SD	T1Mean± SD	Main effect of TIME (F, df)	*P*	ηp²	Covariate (rehabilitation days) p
Barthel Index	29	51.72±23.04	60.00±24.93	F_(1, 27)_ = 18.802	**< .001**	.411	.246
10MWT (s)	12	27.79±24.58	21.87±20.14	F_(1, 10)_ = 1.777	.212	.151	.036
9HPT – paretic arm (s)	14	33.99±19.69	31.56±12.47	F_(1, 12)_= 0.698	.420	.055	.938
9HPT – non-paretic arm (s)	27	23.97±9.03	22.41±7.25	F_(1, 25)_ = 3.717	.065	.129	.768

﻿Significant differences are marked in bold (<.05)

### Correlation analyses

Means and standard deviations across the collection data period (10 days) for each sleep variable were reported in the Supplementary Materials (Table S1).

Correlation analyses were conducted to investigate the association between subjective or actigraphic sleep measures and functional outcomes at the end of data collection. For completeness, correlations between sleep measures and motor outcomes (10MWT and 9HPT for both the paretic and non-paretic arm) are reported in the Supplementary Materials (Table S1).

For functional independence, a significant negative association emerged between the Barthel Index at T1 and both actigraphy-derived sleep duration and self-reported time in bed. Specifically, TBT (*r* = − 0.409, *p*=.028) and TST (ρ=−0.414, *p*=.025) were negatively correlated with Barthel Index at the end of data collection. This means that higher time in bed and sleep duration were both related to poorer functional independence at T1. No other subjective or objective sleep parameters showed significant associations with Barthel Index.

Correlation analyses were reported in Table 3.

**Table 3:** Correlations between subjective (self-reported) and actigraphy-based sleep measures and functional and motor outcomes at T1. Values represent Pearson’s correlation coefficient (*r*) or Spearman’s rank correlation coefficient (ρ), according to data distribution assessed with the Shapiro–Wilk test

Sleep measures/Outcomes	Barthel Index T1 (*N* = 29)
TST – subjective	*r* = − 0.244, *p* = .203
SEI – subjective	*r* = 0.213, *p* = .267
WASO – subjective	ρ = −0.041, *p* = .834
NOA – subjective	*r* = 0.264, *p* = .167
SOL – subjective	ρ = −0.250, *p* = .190
TBT – subjective	*r* = − 0.409, ***p = .028***
TST – actigraphy	**ρ = −0.414**, ***p = .025***
SOL – actigraphy	ρ = −0.050, *p* = .799
SEI – actigraphy	*r* = − 0.279, *p* = .143
WASO – actigraphy	*r* = 0.116, *p* = .550
NOA – actigraphy	ρ = 0.203, *p* = .290

Significant correlations in bold (*p* < .05)

*SOL *Sleep Onset Latency, *TST *Total Sleep Time, *SEI *Sleep Efficiency Index, *TBT *Total Bed Time, *WASO *Wakefulness After Sleep Onset, *NOA *Number of Awakenings

### Moderation analyses

Exploratory, hypothesis-driven moderation analyses were conducted to investigate whether baseline functional status moderated the associations between sleep parameters and functional outcome at T1. These analyses were restricted to sleep variables that showed significant unadjusted associations with the Barthel Index at T1 and were guided by the hypothesis that baseline functional independence might modify these associations.

Specifically, because the Barthel Index was the only functional outcome showing a significant improvement from the start of data collection (T0) to the end of data collection (T1), and because only actigraphy-derived TST and self-reported TBT were significantly correlated with the Barthel Index at T1, moderation analyses were restricted to these two sleep parameters. In both models, the dependent variable was the Barthel Index at T1, the predictor was the sleep parameter (TST or TBT), the moderator was functional status at T0 (Barthel Index), and the number of rehabilitation days completed before the beginning of the monitoring period was included as a covariate.

The first moderation model tested whether functional status at T0 moderated the association between actigraphy-derived TST and functional status at T1 (Barthel Index). The overall moderation model was significant (R² = 0.903, adjusted R² = 0.886, F_(4,24)_ = 55.61, *p* < .001). After adjustment for rehabilitation days and inclusion of baseline Barthel Index as the moderator, the interaction between actigraphy-derived TST and the Barthel Index at T0 was significant (estimate = 0.0033, SE = 0.0012, 95% CI [0.0007, 0.0059], *p* = .014), indicating a moderation effect of initial functional status on the relationship between sleep duration and functional outcome. The covariate representing rehabilitation days was not statistically significant (*p* = .130). Simple slope analyses showed that the effect of TST on the Barthel Index at T1 was significant only at high levels of functional status (+ 1 SD; estimate = 0.129, *p* = .015), whereas it was not significant at average levels (estimate = 0.053, *p* = .082) or in patients with low functional status at T0 (− 1 SD; estimate = − 0.023, *p* = .460). This pattern indicates that longer sleep duration was associated with better functional independence at T1 only in patients with higher levels of functional autonomy at the start of the observation period.

**Fig. 2:**
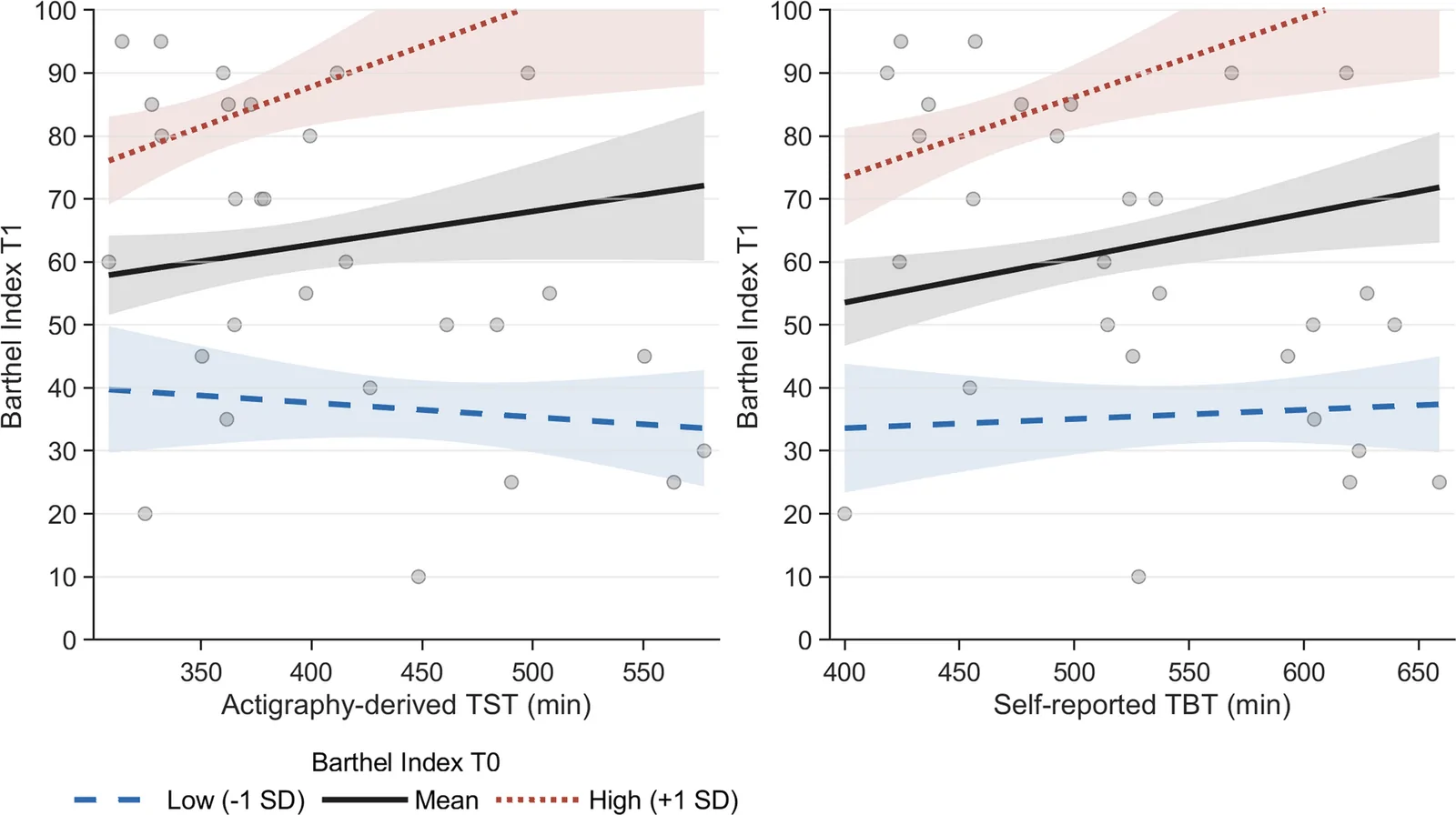
Moderation analyses of the associations between sleep parameters and functional outcome according to baseline functional status. The left panel shows the Barthel Index at T1 as a function of actigraphy-derived total sleep time (TST) at low (− 1 SD), mean, and high (+ 1 SD) levels of Barthel Index at T0. The right panel shows the Barthel Index at T1 as a function of self-reported total bed time (TBT) at low (− 1 SD), mean, and high (+ 1 SD) levels of the Barthel Index at T0. Lines represent model-predicted values, shaded areas represent 95% confidence intervals, and grey circles represent observed values. Models were adjusted for the number of rehabilitation days before sleep monitoring. The y-axes are restricted to the theoretical range of the Barthel Index (0–100)

The second moderation model examined whether functional status at T0 moderated the association between self-reported TBT and functional status at T1 (Barthel Index). The overall moderation model was significant (R² = 0.908, adjusted R² = 0.892, F_(4,24)_ = 59.01, *p* < .001). After adjustment for rehabilitation days and inclusion of baseline Barthel Index as the moderator, the interaction between TBT and the Barthel Index at T0 was significant (estimate = 0.0024, SE = 0.0011, 95% CI [0.0002, 0.0047], *p* = .037), indicating that the relationship between time spent in bed and functional independence at T1 varied as a function of initial functional status. The covariate representing rehabilitation days was not statistically significant (*p* = .126). Simple slope analyses showed that the association between TBT and the Barthel Index at T1 remained significant at average (estimate = 0.071, *p* = .011) and high (+ 1 SD; estimate = 0.127, *p* = .006) levels of baseline functional status, whereas it was not significant at low levels (− 1 SD; estimate = 0.015, *p* = .613). These findings indicate that longer time spent in bed was associated with higher Barthel Index scores at T1 in patients with average-to-high baseline functional independence, whereas no significant association was observed in patients with lower baseline functional independence. The significant moderation effects are illustrated by plotting simple slopes at − 1 SD, mean, and + 1 SD of the Barthel Index at T0 (Fig. 2, panels A and B).

## Discussion

Our exploratory study showed that in unadjusted analyses, longer TST and greater TBT were associated with lower functional independence at the end of the observation period. However, these associations depended on patients’ initial functional status: after accounting for functional independence at T0, longer actigraphy-derived sleep duration and time in bed were positively associated with functional outcome only among patients with higher baseline functional status.

At first glance, the negative unadjusted associations between longer sleep duration, greater self-reported time in bed, and lower Barthel Index scores at T1 may appear counterintuitive, given the established role of sleep in neural plasticity, learning, and memory consolidation [1, 2]. However, in the context of subacute stroke rehabilitation, longer sleep duration or extended time in bed should not necessarily be interpreted as marker of more restorative sleep or more favorable functional outcome. In clinically vulnerable populations, prolonged sleep may instead reflect greater disability, reduced daytime activation, inactivity, or broader medical and neurological burden [8, 25]. Accordingly, the negative simple correlations observed here suggest that, in some patients, longer nocturnal sleep and greater time spent in bed may primarily indicate reduced functional capacity rather than beneficial recovery-related processes per se [13, 26].

The most relevant finding of the present study is that this relationship was not uniform across patients. Although exploratory and data-driven, moderation analyses indicated that the associations between sleep parameters and Barthel Index at T1 differed according to baseline functional independence. Specifically, the association between actigraphy-derived TST and Barthel Index at T1 was positive only at higher levels of functional independence at T0, whereas the association between self-reported TBT and Barthel Index at T1 was positive at average and higher baseline levels. Neither correlation was significant among patients with lower functional status at T0. Taken together, these findings suggest that baseline functional status is not merely a background characteristic, but a potential factor in the interpretation of post-stroke sleep parameters. In other words, the clinical meaning of longer sleep duration and greater time in bed may depend on the patient’s initial functional condition.

This finding may help explain why the literature on sleep duration after stroke has often been heterogeneous and difficult to interpret [6, 10]. If patients with markedly different baseline functional profiles are considered together, longer sleep may appear globally associated with poorer outcome, largely because more impaired individuals tend to spend more time in bed or sleep longer while also having worse functional prognosis. However, once baseline functional status is explicitly taken into account, a more differentiated pattern emerges. As previously mentioned, prolonged sleep and extended time in bed may primarily index greater disability, reduced mobilization, and increased clinical burden in patients with lower functional independence. Nevertheless, in patients with better preserved functional status, longer sleep or longer time in bed may have a different clinical meaning in the context of processes related to sleep-dependent consolidation and restoration [1, 2]. Importantly, the present results do not demonstrate that longer sleep causes better outcome in these patients. Rather, they are consistent with the possibility that the association between sleep duration and later functional outcome is conditional on baseline functional status and should therefore not be interpreted uniformly across all stroke survivors.

This interpretation is broadly in line with previous evidence showing that post-stroke sleep disturbances are associated with poorer functional outcome and reduced participation in rehabilitation, while also highlighting the complexity of sleep-related markers in neurological populations [8]. Sleep complaints, fragmented sleep, insomnia symptoms, and broader sleep–wake disturbances are highly prevalent after stroke and have been linked to less favorable outcomes [7, 9]. At the same time, recent evidence suggests that specific sleep alterations in the subacute phase may be differentially associated with later functional outcome. In particular, reduced TST, insomnia symptoms, restless legs syndrome, and lower REM sleep have been associated with poorer 3-month functional outcomes after ischemic stroke [27]. Taken together, these findings are consistent with the view that sleep parameters in post-stroke rehabilitation may reflect different aspects of the patient’s clinical and functional condition, depending on the broader context in which they are observed [8, 27].

These findings should be interpreted in light of the functional changes observed during rehabilitation. Barthel Index scores significantly increased over the monitored period, indicating improved functional independence. As the primary outcome, the Barthel Index captures a broad range of everyday abilities relevant to stroke rehabilitation [28]. Performance-based motor tests were included as complementary exploratory measures to examine whether sleep–outcome associations extended to more specific motor domains. However, these measures did not show significant pre–post changes after controlling for previous rehabilitation days. This is not unexpected, as functional independence and motor performance reflect partly distinct dimensions of post-stroke functioning and may differ in sensitivity over a short observation period [29]. Moreover, motor tests may be affected by floor effects in heterogeneous inpatient samples, particularly when some patients are unable to complete the task at one or both assessments.

Overall, our results also have potentially relevant clinical implications. They suggest that sleep assessment during inpatient stroke rehabilitation should not be limited to a generic classification of “more” or “less” sleep as beneficial or detrimental. Instead, sleep duration and time in bed should be interpreted considering the patient’s baseline functional condition. In more severely impaired patients, prolonged sleep or extended time in bed may warrant clinical attention as possible indicators of inactivity, greater dependency, or more complex medical burden [8, 13]. This supports a more individualized and severity-aware approach to sleep assessment in neurorehabilitation settings [6, 9], although these potential clinical implications should be interpreted cautiously given the exploratory nature of the study.

Several limitations should be acknowledged. First, the relatively small sample size limited statistical power and the number of covariates that could be reliably included in the analyses. Several clinical and stroke-related characteristics were available and descriptively reported, including stroke type, lesion hemisphere, time since stroke, and rehabilitation exposure; however, their simultaneous inclusion as additional covariates in the moderation models would have resulted in overparameterized and potentially unstable models. Moreover, systematic descriptive information on some other potentially relevant clinical confounders, particularly comorbidities and medication use, was not available for the entire sample and could therefore not be comprehensively reported or incorporated into the statistical models. The clinical heterogeneity of the sample in terms of stroke type, time since stroke, and rehabilitation stage may have further contributed to variability in sleep patterns and functional outcomes. Consequently, residual confounding cannot be excluded. Post-stroke sleep–wake patterns and daytime sleepiness may vary according to lesion location and stroke characteristics and may also be associated with depressive symptoms, fatigue, cognitive impairment, pain, and medical comorbidities [30–32]. In addition, medications commonly prescribed after stroke may affect sleep architecture, sleep continuity, arousal, and daytime activity; for example, antidepressants and sedative agents may alter sleep–wake patterns, while other treatments for stroke-related comorbidities may contribute to insomnia, daytime fatigue, or sleepiness [31].

Second, although actigraphy estimates sleep indirectly from motor activity and may therefore misclassify prolonged immobility as sleep, it is a recognized method for monitoring sleep–wake patterns over multiple consecutive days and has been widely applied in stroke rehabilitation populations [14, 33, 34]. In the present study, this potential bias was minimized by placing the device on the non-paretic arm, consistent with previous stroke studies [14, 34]. Moreover, participants were able to engage in the rehabilitation and monitoring protocol and did not present profound bilateral upper-limb immobility. Nevertheless, some degree of sleep–wake misclassification cannot be entirely excluded. Moreover, it should be considered that actigraphy does not assess sleep architecture or quantitative EEG features that may be more directly linked to rehabilitation-related gains [27, 35]. Additionally, although patients with a previous diagnosis of obstructive sleep apnea were excluded and participants underwent overnight pulse oximetry screening according to routine clinical practice, systematic polysomnography or respiratory polygraphy was not performed in all participants. Therefore, undiagnosed sleep-disordered breathing cannot be completely excluded and may have influenced both sleep parameters and functional outcome. Third, given the observational design of the study, no causal relationship between sleep parameters and functional outcome can be inferred. The observed findings should therefore be interpreted exclusively as associations between sleep characteristics and later functional status within the monitored rehabilitation interval. Another limitation is the lack of information on pre-stroke sleep characteristics, since it was not possible to determine whether the observed sleep parameters were entirely attributable to stroke or partly reflected pre-existing individual sleep habits. Future prospective studies should include information on habitual pre-stroke sleep patterns, including retrospective reports from patients or caregivers when feasible, to better distinguish pre-existing sleep characteristics from stroke-related changes. Finally, several sleep variables were examined in exploratory correlation analyses without formal correction for multiple comparisons. Although this decision was made to preserve sensitivity in the context of the small sample, it increases the possibility of type I error. The selection of variables for the moderation analyses was also based on these preliminary associations.

Despite these limitations, this exploratory study contributes to the growing literature on sleep and stroke rehabilitation by suggesting that baseline functional status may be relevant to the interpretation of the association between sleep and functional outcome. The results indicate that sleep duration and time in bed may have different clinical meanings depending on the patient’s initial level of independence. These findings should be considered preliminary and hypothesis-generating rather than confirmatory. Given the relatively small and clinically heterogeneous sample, the moderation effects should be interpreted with caution and require replication in larger, adequately powered cohorts before firm conclusions can be drawn. Future studies with larger and more homogeneous samples, longer follow-up periods, and more comprehensive clinical characterization are needed to better characterize the relationship between sleep parameters, clinical severity, and functional outcomes across different stages after stroke [6, 8, 27, 35].

## Supplementary Information

Below is the link to the electronic supplementary material.

Supplementary material (33.8 KB)

## Data Availability

The datasets generated and/or analyzed during the current study are not publicly available due to ethical and legal restrictions related to participant privacy. Requests for access to de-identified data should be directed to the Principal Investigator, Prof. Marco Iosa, and will be considered subject to appropriate ethical, legal, and institutional approvals.
