# Supplementary material for: Does sleep matter for recovery after stroke? Functional severity shapes the association between sleep and functional outcome during inpatient stroke rehabilitation
Source: Neurol Sci. 2026 Oct 1;47(10):829. doi: 10.1007/s10072-026-09374-w (PMC13630849; doi:10.1007/s10072-026-09374-w)
Supplement: Supplementary file 1 [file 10072_2026_9374_MOESM1_ESM.docx]

**Supplementary Materials**

***Measures***

*Participation during rehabilitation period*

The  Pittsburgh Rehabilitation Participation Scale (PRPS) was administered throughout the study to ensure that the level of engagement remained stable across the rehabilitation period. It is an observational scale designed to investigate the degree of patient participation in physical and occupational rehabilitation therapy, consisting of a single item rated by the clinician, with six possible scores: (1) no participation, if the patient refused the entire session; (2) poor, if the patient refused or did not participate in at least half of the session; (3) fair, if participation occurred in most or all exercises but without maximal effort or requiring encouragement to complete them; (4) good, if the patient participated in all exercises with good effort and completed most of them; (5) very good, if the patient participated in all exercises with maximal effort and completed all of them while passively following instructions; and (6) excellent, if the patient participated in all exercises with maximal commitment, completed all exercises, and showed active interest in the rehabilitation session.

*Outcome measures*

The assessment of rehabilitation outcomes was conducted using the Barthel Index, the 10-Meter Walk Test (10MWT), and the 9-Hole Peg Test (9HPT).

- Barthel Index: It is a physical assessment tool designed to determine the degree of functional independence in relation to the main activities of daily living (ADL), such as feeding, dressing, personal hygiene management, toileting, and climbing stairs. Item scores vary according to the activity assessed, and the total score ranges from 0, indicating complete dependence, to 100, indicating complete functional independence.
- 10-Meter Walk Test (10MWT): This is a tool used to assess walking speed over a short distance, specifically to determine participants’ functional mobility, gait, and vestibular function. The participant walks a predetermined distance (10 meters) while the time is measured with a stopwatch. The 10MWT was used to identify changes in gait speed following therapeutic interventions.
- 9-Hole Peg Test (9HPT): This is a standardized test that assesses manual dexterity of both the paretic and non-paretic upper limbs. The participant is asked to pick up pegs from a container, place them into the holes of a board, and then remove them and return them to the container as quickly as possible. The time required to complete the task constitutes the final score, expressed in seconds.

*Sleep pattern*

- Pittsburgh Sleep Quality Index (PSQI): This self-report questionnaire assesses subjective sleep quality over the previous month, and it was administered at T0. It comprises 19 items across seven components (e.g., quality, latency, duration, and efficiency) scored on a 0–3 Likert scale. A global score above 5 indicates poor sleep quality.
- Sleep diaries: Participants completed a standardized sleep diary every morning within 30 minutes of awakening, with the assistance of a researcher to ensure data accuracy and compliance. The diary collected information on subjective characteristics of the preceding night's sleep. Specifically, participants answered the following questions: a) bedtime: “What time did you go to bed?”; b) lights-out time: “What time did you turn off the lights with the intention of going to sleep?”; c) sleep-onset latency: “Approximately how many minutes did it take you to fall asleep?”; d) number of awakenings: “How many times did you wake up during the night?”; e) duration of nocturnal awakenings: “Approximately what time did your first/second/third/fourth awakening begin and end?”; f) wake after sleep onset: “Approximately how many minutes in total did you remain awake during the night after initially falling asleep?”; g) total sleep time: “Approximately how long did you sleep in total? Please report the duration in hours and minutes.”; h) final awakening time: “What time did you wake up for the final time this morning?”; i) subjective sleep quality: “Overall, how would you rate the quality of your sleep last night?”.

Total sleep time (TST), sleep onset latency (SOL), and the number of awakenings were obtained directly from participants' reports. Total bed time (TBT) was calculated as the interval between the reported in-bed and out-of-bed times, and sleep efficiency index (SEI) was calculated as the ratio of TST to TBT, expressed as a percentage.

- Actigraphy: Objective sleep-wake parameters were recorded using the GT9X Link actigraph (ActiGraph, Pensacola, FL). The device was worn continuously on the wrist of the non-paretic arm to avoid underestimation of movement due to motor impairment. The actigraph was applied to patients able to participate in the rehabilitation and monitoring procedures, rather than to individuals with profound bilateral upper-limb immobility. Only nocturnal sleep periods were included in the analyses; daytime naps were not analyzed. Participants were required to have at least seven valid recording nights. Of the 29 participants with actigraphy data, 24 had 10 valid nights, three had nine valid nights, one had eight valid nights, and one had seven valid nights. Missing nights were not imputed; for each participant, actigraphy-derived sleep parameters were averaged across all available valid nights. Data were sampled using a triaxial accelerometer and processed via ActiLife 6 software. Activity count data were collected at 30 Hz and were downloaded and processed in 60-s epochs. For each recording night, the diary-reported in-bed and out-of-bed times were manually entered into ActiLife to define the nocturnal rest interval. Sleep–wake scoring was then performed only within this interval using the validated Cole–Kripke algorithm, which classified each 60-s epoch as sleep or wake. Based on this scoring, the software automatically computed the objective sleep parameters. Consequently, actigraphy-derived TBT was defined by the same diary-reported in-bed and out-of-bed times used to establish the nocturnal rest interval.

Then, the following objective parameters were calculated:

- TST: The total number of minutes scored as “asleep”.
- SOL: The period between bedtime and the first epoch of sleep.
- SEI: The ratio between TST and TBT (%).
- Wake After Sleep Onset (WASO): The total number of minutes the subject was marked awake after sleep onset occurred.
- Number of Awakenings (NOA): The number of different awakening episodes as scored by the algorithm.

**Results of control analyses**

To verify that good and poor sleepers did not differ in clinical, functional, motor or sleep-related characteristics, a series of independent-samples *t* tests were performed comparing the two groups (defined according to the sleep classification criterion) on scores at T0, T1 and change scores, as well as on participation and sleep variables. No significant between-group differences were observed for any functional outcome (Barthel Index at T0 and T1, Barthel Effectiveness as normalized index of improvement), motor performance (10MWT and 9HPT, paretic and non-paretic arm, and related T0/T1 ratio as additional indicator of improvement), rehabilitation participation (PRPS), or subjective and actigraphy-derived sleep measures (all *p* > 0.13). These results indicate that good and poor sleepers were comparable across clinical severity, functional and motor status, participation in rehabilitation, and sleep characteristics.

Furthermore, the repeated-measures ANOVA performed on the Pittsburgh Rehabilitation Participation Scale (PRPS) scores showed that patients’ participation in rehabilitation remained stable across the 10-day target period (mean PRPS = 5.14, SD = 1.24; *F* = 0.84, p = .52). A mean PRPS value of approximately 5 corresponds to a very good level of active participation in rehabilitation sessions, suggesting that no systematic changes in participation occurred during the monitoring period.

**Table S1. Statistical comparisons (unpaired t-test) between good and poor sleepers**

| Variable | Good Sleepers (mean ± SD) | Poor Sleepers (mean ± SD) | t (df) | p |
| --- | --- | --- | --- | --- |
| Barthel Index T0 | 57.00 ± 30.20 | 48.95 ± 18.60 | 0.89 (27) | .381 |
| Barthel Index T1 | 67.00 ± 31.90 | 56.32 ± 20.40 | 1.10 (27) | .281 |
| Barthel Effectiveness | 27.67 ± 32.30 | 16.82 ± 16.18 | 1.22 (27) | .235 |
| 9HPT paretic arm T0 (s) | 26.92 ± 12.91 | 37.91 ± 22.32 | −1.00 (12) | .337 |
| 9HPT non-paretic arm T0 (s) | 23.87 ± 6.96 | 23.71 ± 9.76 | 0.05 (27) | .963 |
| 9HPT paretic arm T1 (s) | 25.35 ± 11.20 | 32.26 ± 12.62 | −1.12 (15) | .281 |
| 9HPT non paretic arm T1 (s) | 24.19 ± 8.98 | 21.52 ± 6.33 | 0.90 (25) | .377 |
| 10MWT T0 (s) | 38.12 ± 35.31 | 20.41 ± 11.13 | 1.26 (10) | .235 |
| 10MWT T1 (s) | 25.90 ± 28.53 | 15.71 ± 6.60 | 0.99 (12) | .342 |
| Ratio 9HPT paretic arm | 1.01 ± 0.20 | 1.11 ± 0.75 | −0.27 (12) | .789 |
| Ratio 9HPT non paretic arm | 1.01 ± 0.30 | 0.95 ± 0.18 | 0.67 (25) | .510 |
| Ratio 10MWT | 0.77 ± 0.15 | 0.86 ± 0.15 | −0.95 (10) | .367 |
| PRPS (mean) | 5.20 ± 0.68 | 5.11 ± 1.47 | 0.18 (27) | .858 |
| SOL – subjective (min) | 24.10 ± 22.66 | 40.92 ± 30.61 | −1.53 (27) | .139 |
| NOA – subjective | 12.05 ± 6.92 | 8.84 ± 6.79 | 1.20 (27) | .240 |
| WASO – subjective (min) | 28.96 ± 26.68 | 31.14 ± 30.82 | −0.19 (27) | .851 |
| TBT – subjective (min) | 531.49 ± 82.72 | 522.07 ± 77.43 | 0.30 (27) | .763 |
| TST – subjective (min) | 478.43 ± 95.70 | 450.01 ± 78.99 | 0.86 (27) | .399 |
| SEI – subjective (%) | 90.00 ± 8.00 | 86.00 ± 9.00 | 1.13 (27) | .269 |
| SOL – actigraphy (min) | 7.78 ± 6.79 | 11.95 ± 10.95 | −1.09 (27) | .283 |
| SEI – actigraphy (%) | 79.69 ± 7.33 | 77.52 ± 8.12 | 0.71 (27) | .486 |
| TST – actigraphy (min) | 423.63 ± 81.47 | 401.39 ± 76.79 | 0.73 (27) | .474 |
| WASO – actigraphy (min) | 99.89 ± 34.53 | 107.42 ± 45.62 | −0.46 (27) | .652 |
| NOA – actigraphy | 134.42 ± 88.54 | 114.43 ± 77.81 | 0.63 (27) | .536 |

*10MWT, 10-Meter Walk Test; 9HPT, 9-Hole Peg Test, PRPS, Pittsburgh Rehabilitation Participation Scale, SOL, Sleep Onset Latency; TST, Total Sleep Time; SEI, Sleep Efficiency Index; TBT, Total Bed Time; WASO, Wakefulness After Sleep Onset; NOA, Number of Awakenings*

**Table S2. Descriptive statistics of subjective (self-reported) and actigraphy-derived sleep measures in the study sample (N = 29). Values are reported as means and standard deviations. Total sleep time (TST), total bed time (TBT), sleep onset latency (SOL), and wake after sleep onset (WASO) are expressed in minutes, where applicable. Sleep efficiency index (SEI) is expressed as a percentage.**

| Variable | N | Mean | SD |
| --- | --- | --- | --- |
| SOL – subjective (min) | 29 | 35.1 | 28.9 |
| TST – subjective (min) | 29 | 460 | 84.5 |
| TBT – subjective (min) | 29 | 524 | 78.1 |
| WASO - subjective (min) | 29 | 30.4 | 29.0 |
| NOA - subjective | 29 | 9.95 | 6.89 |
| SEI – subjective (%) | 29 | 87.1 | 8.00 |
| SOL – actigraphy (min) | 29 | 10.5 | 9.80 |
| TST – actigraphy (min) | 29 | 409 | 77.7 |
| WASO – actigraphy (min) | 29 | 105 | 41.6 |
| NOA - actigraphy | 29 | 121 | 80.7 |
| SEI - actigraphy (%) | 29 | 78.3 | 7.80 |

*SOL, Sleep Onset Latency; TST, Total Sleep Time; SEI, Sleep Efficiency Index; TBT, Total Bed Time; WASO, Wakefulness After Sleep Onset; NOA, Number of Awakenings*

**Table S3. Correlations between subjective (self-reported) and actigraphy-based sleep measures and motor outcomes at T1.**

Values represent Pearson’s correlation coefficient (*r*) or Spearman’s rank correlation coefficient (ρ), according to data distribution assessed with the Shapiro–Wilk test. 10MWT T1 and 9HPT (paretic and non-paretic arm) represent completion time in seconds, with higher values indicating poorer performance. Sample size varies across outcomes because of missing data.

| Sleep measures/Outcomes | 10MWT T1 (N=14) | 9HPT T1 paretic arm (N=17) | 9HPT T1 non paretic arm (N=27) |
| --- | --- | --- | --- |
| TST – subjective | ρ = 0.319, p = .267 | r = −0.009, p = .973 | r = −0.167, p = .406 |
| SEI – subjective | ρ = 0.253, p = .382 | r = 0.098, p = .709 | r = −0.052, p = .799 |
| WASO – subjective | ρ = −0.130, p = .660 | ρ = −0.181, p = .507 | ρ = 0.133, p = .507 |
| NOA – subjective | ρ = 0.101, p = .731 | r = −0.062, p = .812 | r = 0.228, p = .254 |
| SOL – subjective | ρ = −0.160, p = .584 | ρ = 0.127, p = .625 | ρ = −0.045, p = .822 |
| TBT – subjective | ρ = 0.169, p = .563 | r = −0.097, p = .711 | r = −0.139, p = .488 |
| TST – actigraphy | ρ = 0.077, p = .797 | ρ = 0.152, p = .559 | ρ = −0.102, p = .612 |
| SOL – actigraphy | ρ = 0.147, p = .616 | ρ = 0.034, p = .898 | ρ = 0.106, p = .599 |
| SEI – actigraphy | ρ = 0.160, p = .584 | r = 0.192, p = .460 | r = 0.041, p = .837 |
| WASO – actigraphy | ρ = 0.059, p = .844 | r = −0.206, p = .427 | r = −0.013, p = .949 |
| NOA – actigraphy | **ρ = 0.604, p = .025** | ρ = 0.140, p = .592 | ρ = 0.268, p = .176 |

*Significant correlations in bold (p < .05)*

*10MWT, 10-Meter Walk Test; 9HPT, 9-Hole Peg Test, SOL, Sleep Onset Latency; TST, Total Sleep Time; SEI, Sleep Efficiency Index; TBT, Total Bed Time; WASO, Wakefulness After Sleep Onset; NOA, Number of Awakenings*

Regarding motor performance, a significant positive association was observed between the number of nocturnal awakenings measured by actigraphy (NOA) and the 10MWT score at the end of data collection (T1; ρ = 0.604, p = .025). Since the 10MWT is expressed in seconds, this finding indicates that a higher number of nocturnal awakenings was associated with slower walking speed and therefore poorer walking performance.
